# Association between employer’s knowledge and attitude towards smoking cessation and voluntary promotion in workplace: a survey study

**DOI:** 10.1186/s12971-017-0149-4

**Published:** 2017-11-14

**Authors:** Man Ping Wang, William Ho Cheung Li, Yi Nam Suen, Ka Ching Cheung, Oi Sze Lau, Tai Hing Lam, Sophia Siu Chee Chan

**Affiliations:** 1 School of Nursing, The University of Hong Kong, Hong Kong, SAR China; 2 Department of Psychiatry, The University of Hong Kong, Hong Kong, SAR China; 3 Department of Asian and Policy Studies, The Education University of Hong Kong, Hong Kong, SAR China; 4 The Lok Sin Tong Benevolent Society, Kowloon, Hong Kong, SAR China; 5 School of Public Health, the University of Hong Kong, Hong Kong, SAR China

**Keywords:** Voluntary, Smoking cessation, Workplace, Chinese, Company

## Abstract

**Background:**

Workplace smoking cessation (SC) intervention is effective in increasing quit rate but little was known about the factors associated with voluntary SC promotion. Comprehensive smoke-free legislation, including banning smoking in all indoor area of workplaces, has been enforced in Hong Kong. This survey investigated the prevalence of company’s compliance with smoke-free legislation and examined the relation between voluntary SC promotion in workplace and employer’s knowledge of and attitude towards smoking and SC.

**Methods:**

Half (50.3%, *n* = 292) of a convenience sample of companies completed a self-administered questionnaire on company’s voluntary SC promotion in the workplace. Factors investigated included company’s characteristics (size, type, and number of smoking employees); employers’ knowledge of smoking, second-hand smoke and SC effects on health; perceived responsibility in assisting employees to quit smoking and smoking prohibition in workplace (smoke free policy). Logistic regression yielded adjusted odds ratio (aOR) for voluntary SC promotion.

**Results:**

A notable proportion of companies (14.7%) showed non-compliance with the smoke free workplace ordinance and only 10% voluntarily promoted SC. Perceived greater negative impact of smoking on the company (adjusted odds ratio[aOR] 1.94, 95% confidence interval [CI] 1.18-3.20) and better knowledge of smoking (aOR 1.40, 95%CI 1.00-1.94) were associated with voluntary SC promotion. Positive but non-significant associations were observed between perceived responsibility of assisting employees to quit, workplace smoke free policy and voluntary SC promotion. Company characteristics were generally not associated with voluntary SC promotion except white collar companies were less likely to promote SC (aOR 0.26, 95% CI 0.08-0.85).

**Conclusions:**

This is the first survey on company’s SC promotion in the Chinese population. A notable proportion of companies was not compliant with the smoke-free workplace ordinance. Employers with a higher level of knowledge and perceived impact of smoking on companies and from blue-collar companies were more likely to promote SC in workplace. The findings inform future workplace intervention design and policy.

**Trial registration:**

The study was retrospectively registered at ClinicalTrials.gov (NCT02179424) dated 27 June 2014.

## Background

Smoke-free workplace policy is effective in reducing cigarette consumption, increasing quit rate, strengthening current tobacco control initiatives and protecting non-smoking employees from secondhand smoke (SHS) exposure [1]. However, the adoption is inconsistently prevalent in Western countries, ranging from 20% in Greece to 54% in the US and 88% in the UK [2, 3]. Similar study is scarce in Asian countries. One study has shown that less than half of the companies in Taiwan adopted smoke-free policy [4]. Regarding the low adoption rate, employers expressed their concerns on the lack of perceived responsibility for maintaining a smoke-free workplace, the insufficient knowledge on the impact of smoking on the company and employees, and the worries about undermining employee’s morale and customer’s satisfaction [5].

Previous study has found that non-smoking employers with positive attitude towards health and higher level of perceived responsibility were more likely to promote SC in workplace voluntarily [2]. Particularly, Chinese employers are less likely to consider assisting employees to quit smoking as their responsibility when compared with their western counterparts [6]. Such difference might be associated with the difference in the progress of tobacco control and smoke-free legislation, social norm of smoking, and attitudes and knowledge of employers on tobacco control in the workplace.

Hong Kong, as the most urbanised and westernised city in China, its smoking prevalence is amongst the lowest in the world (10.5% in 2015) as various tobacco control measures have been enforced since 1982 [7]. The measures include imposing heavy tobacco tax (70% of the retail price), banning smoking in most indoor and public places, completely banning tobacco advertisement, enlarging the pictorial warnings (85% of the cigarette pack) and providing free SC services [7, 8].

In the formative component of a workplace SC project organised by Lok Sin Tong Benevolent Society, Kowloon (LST) commissioned by the Tobacco Control Office of Department of Health (Hong Kong SAR), we surveyed on company’s characteristics and employer’s knowledge, attitude and practice towards SC promotion. This study investigated the prevalence of company’s compliance with smoke-free workplace ordinance and the relation between voluntary workplace SC promotion and employer’s knowledge and attitude towards smoking and SC.

## Methods

### Study design

This cross-sectional survey was conducted in 2012. A self-administered questionnaire in Chinese was mailed to 580 companies which were selected from the list of the Caring Company Scheme 2012 organised by the Hong Kong Council of Social Services (largest database of companies in Hong Kong). The questionnaire was required to be completed by adult (aged ≥18) staff who was at managerial or above level including supervisors, managers and employers (hereafter as “employers”).

### Measurements

The questionnaire collected information on (1) company’s characteristics including size, industry type and the number of smoking employees and (2) employer’s knowledge, attitude and practice (KAP) in SC promotion. Companies size was classified into small (≤10 employees), medium (11-100) and large (≥100) by the number of employees. Company’s industry type was categorised as blue-collar (construction, transportation food/restaurants & property management), white-collar (insurance, finance/bank, education & information technology) or others (service, e.g., charity & hospital). Employer’s *knowledge* in SC promotion was assessed through seven items concerning the positive/negative consequences of SC, the use of nicotine replacement therapy (NRT) and SHS. The items were regarded as most commonly misunderstood concept of smoking, cessation and SHS, generated by a panel of experts in the research team based on their substantial SC promotion experience. Similar items have been used in previous studies to test the knowledge of the personnel who engaged in SC promotion programme [9, 10]. Employers rated the items on a 4-point Likert scale ranging from 1 (strongly agree) to 4 (strongly disagree) and were considered as having a correct response if he agreed/ strongly agreed to a correct statement or disagreed/ strongly disagreed with an incorrect statement. Correct responses to these seven items were summed to derive a score of 0-7 in which higher scores indicated a higher level of knowledge. Two sub-scales were designed by the authors to assess employer’s *attitude* towards SC promotion, that including their (1) perceived responsibility towards SC promotion and smoke-free policy in the workplace and (2) perceived impact of smoking on the company. The former was measured using a dichotomous question: “Whether employers have the responsibility to assist their employees to quit smoking?” (yes or no). The latter was measured by self-rating their perception of how smoking impact on company’s productivity, image, working environment, environment outside the workplace and consumer perceived quality of service, using a 4-point Likert scale ranging from 1 (no impact) to 4 (severe impact). The scores of 3 or above (somewhat or severe impact) were categorized as “have perceived the negative impact of smoking on the company”. Employer’s *practice* in SC promotion was assessed by a set of questions related to any voluntary SC promotion in the past 12 months reported by the employers. The promotion could be in any means to promote SC and assist smoking staff to quit, ranging from disseminating information of the available SC services to offering incentives and/or interventions for cessation. Meanwhile, adoption of smoke-free policy in workplace was indicated by four levels of smoking prohibition in the workplace: “very strict”, “strict”, “less strict” and “not strict at all”.

### Statistical analysis

All analyses were performed using Stata version 13.0. Logistic regression was used to yield adjusted odds ratios (aOR) for voluntary SC promotion in relation to company size, company type, smoke-free policy, employers’ perceived impact of smoking on the company, knowledge of smoking, SHS and perceived responsibility to assist employees to quit smoking. A *p*-value <0.05 denoted statistical significance.

## Results

A total of 292 (50.3% of 580 companies) completed and returned the valid questionnaires for data analysis. Companies of different sizes were included equally: small (35.6%), medium (30.1%) and large (34.3%) (Table 1) and classified as blue-collar (32.1%), white-collar (28.2%) and others (39.7%). A notable proportion of companies (14.7%) showed non-compliance with the smoke-free ordinance in workplace. More than half (52.4%) reported having smoking employees in the company, but only one-fourth (24.1%) agreed that they were responsible for assisting employees to quit smoking. Among a few (10.0%) that had ever promoted SC voluntarily in workplace, most delivered SC messages to their employees via posters, notices or leaflets. Many employers (about 90%) were not aware of the negative consequences of smoking on mortality. Over one-third (37.7%) thought that SHS is less harmful than outdoor air pollution and a half (47.8%) were not aware that NRT can increase the quit rate.

**Table 1:** Company’s and employer’s characteristics (*N* = 292)

Company’s characteristics	n (%)
Company size	
Small (*N* = 1-10)	104 (35.6)
Medium (*N* = 11-100)	88 (30.1)
Large (*N* = 100+)	100 (34.3)
Company type^a^	
Blue-collar	92 (31.5)
White-collar	81 (27.7)
Others	119 (40.8)
Having smoking employee	153 (52.4)
Having strict prohibition smokefree policy	249 (85.2)
Employers perceived responsible for assisting employees to quit smoking	70 (24.0)
Provision of assistance to help smoking staff to quit last year	29 (9.9)
Messages (Poster, notice or leaflet)	22 (75.9)
Health talks or enquiries	7 (24.1)
Referral to smoking cessation service (Department of Health)	5 (17.2)
Respondent’s characteristics	
Age (yrs.; mean ± SD, range)	37.4 ± 10.9 (18-85)
Position	
Employer (Owner of the company)	56 (19.2)
Manager	119 (40.8)
Supervisor	117 (40.1)
Perceived impact of smoking on company(0-5; mean ± SD)^b^	3.30 ± 1.85
Productivity	151 (55.7)
Corporate image	195 (70.9)
Work environment	220 (79.4)
Environment outside workplace	215 (78.8)
Customer’s evaluation of service quality	182 (67.4)
Knowledge of smoking and second-hand smoke (0-7; mean ± SD)^c^	3.42 ± 1.67
Quitting is too late if smoked for years	212 (76.3)
Quitting may harm health of elderly smokers and quitting is not necessary	219 (79.4)
Using low-tar cigarettes is a safe alternative to quitting	200 (72.7)
Nicotine gum and patches increase quit rate	132 (47.8)
1 out of 20 smokers will be killed by smoking	34 (12.4)
Preventing children and adolescent smoking is the most important way to reduce smoking-attributable mortality	29 (10.5)
Second-hand smoke is less harmful than outdoor air pollution	172 (62.3)

^a^Top 5 industries (%), in term of the number of companies involved in this study, were Finance/ Bank (11.2%), Information Technology (9.1%), Property Management (8.4%), Food service/ Restaurants (5.6%), Education (4.9%)

^b^Respondent scored 1 point if he/she perceived “somewhat impact” or “severe impact” on the 5 items listed below; the total score ranged from 0 to 5

^c^Respondent scored 1 point if he/she gave a correct answer to each of the listed items; the total score ranged from 0 to 7

Voluntary SC promotion was significantly and positively associated with employer’s perceived impact of smoking on the company (adjusted odds ratio [aOR] 1.94, 95% confidence interval [CI] 1.18-3.20) and knowledge of smoking and SHS (aOR 1.40, 95% CI 1.00-1.94) and negatively associated with being white-collar company (aOR 0.26, 95% CI 0.08-0.85) (Table 2). The perceived responsibility for assisting employees to quit smoking (aOR 1.65, 95% CI 0.65-4.19) and company’s smoke-free policy (aOR 1.25, 95% CI 0.14-11.1) were positively but non-significantly associated with voluntary SC promotion. Company size was not associated with voluntary SC promotion.

**Table 2:** Association between company’s characteristics, employer’s knowledge and attitude and voluntary smoking cessation (SC) promotion in workplace

		Voluntary SC promotion	
	n (%)/ mean ± SD	Crude OR (95% CI)	Adjusted OR (95% CI)^a^
Knowledge of smoking	4.39 ± 1.20	1.59 (1.15-2.21)**	1.40 (1.00-1.94)*
Perceived impact	4.62 ± 0.82	2.17 (1.34-3.49)**	1.94 (1.18-3.20)**
Perceived responsibility			
No	8.2	1.00	1.00
Yes	17.1	2.32 (1.05-5.15)*	1.65 (0.65-4.19)
Smokefree policy			
No	5.6	1.00	1.00
Yes	10.2	1.93 (0.25-15.1)	1.25 (0.14-11.1)
Company size			
Small/Medium	7.9	1.00	1.00
Large	14.1	1.92 (0.89-4.16)	2.10 (0.85-5.14)
Company type			
Blue collar	18.7	1.00	1.00
White collar	4.9	0.23 (0.07-0.70)*	0.26 (0.08-0.85)*
Others	7.1	0.33 (0.14-0.81)*	0.45 (0.16-1.25)

*OR* odds ratio, *CI* confidence interval

*p < 0.05, **p < 0.01

^a^Mutually adjusted for each other

## Discussion

The present study is the first to examine the factors that associated with the adoption of voluntary workplace SC promotion in Chinese. In this study, a quarter of employers (24.0%) perceived the responsibility to assist employees to quit smoking that is lower than the Western (39.0%) and other Asian (38%) countries [6]. The low adoption of voluntary SC promotion may be associated with employer’s insufficient knowledge of smoking (i.e., most employers did not realise the high risk of tobacco-induced mortality) and underestimation of the negative impact of smoking on the company, specifically in the aspect of productivity and customer satisfaction. Indeed, previous studies have reported the association between smoke-free workplace and improved business outcomes such as the number of customers or sales [11, 12]. While employer’s roles in tobacco control and health promotion in workplace have been discussed in several company surveys in the US [2, 13], our finding suggested that the adoption can be predicted by employer’s perceived impact of smoking on the company and their knowledge of smoking and SHS and future interventions shall target on these perspectives.

Workplace smoking has been comprehensively banned in Hong Kong since 2007, but we found that a remarkable proportion of companies (14.7%) was not compliant with the legislation, that is similar to other countries (e.g. Ireland 14%) [14]. The compliance with the smoke-free workplace ordinance is a concern worldwide. The possible explanation on the low compliance in Hong Kong might be due to the deficiencies of the workplace smoke-free ordinance regarding coverage, surveillance and penalty. For instance, regular inspections or blitz operations conducted by the enforcement department only constitutes a small proportion of smoking offences in workplace. In 2014, only 8027 fixed penalty notices or summonses were issued among 29,000 inspections [15]. As the ordinance does not cover outdoor workplaces of the companies, nonsmoking employees and pedestrians are still exposed to SHS [16]. The situation may be even worse in Hong Kong due to its dense population and narrow pavements [17–19]. Banning smoking at outdoor workplaces and the entrances of buildings and shops (within 3 to 9 m) should be considered [20, 21].

Implementation of smoke-free workplace policies may encourage employers to promote SC by denormalising smoking [22]. Establishing non-smoker hiring policies can be one of the measures contributing to motivate smokers to quit and healthier staff [1, 23], regardless the concerns about discrimination against smokers who are mostly in the lower socioeconomic group. Regarding the intervention to motivate quitting, most employers only adopted simple voluntary SC strategies such as posting notices of “smoking is prohibited”. Other effective SC interventions such as providing incentives, time and psychological support for smoking employees to attend SC services are needed [24–29].

Contrary to the observation in the US and Taiwan, large and blue-collar companies in the present study were more likely to promote SC [2, 4, 13]. Large companies are led by written regulations and more likely to comply with the smoke-free workplace ordinance while small/medium companies were more likely to rely on informal communication and to respect individual freedom of smoking [2]. Our finding that SC promotion was more likely to happen in blue collar companies might be due to the higher smoking prevalence in this group in Hong Kong. Notably, the need for SC promotion in the white-collar companies may be neglected. The result suggested that the design of SC promotion activities should consider the company size and type.

This study has several limitations. First, the convenience sampling method may compromise the representativeness of our sample to the population. Specifically, it was likely that the employers who agreed to join our study were more aware of the smoking issues. Thus, the prevalence of smoke-free policy in the workplace might be overestimated. Second, the cross-sectional method limited the casual inference about the associations of employer’s perceived responsibility for assisting employees to quit smoking and the enforcement of smoke-free policies in the company with employer’s SC promotion. Finally, the study relied on self-reported data, which was subjected to response bias.

## Conclusions

This study investigated company’s SC promotion in the Chinese population. A notable proportion of companies was not compliant with the smoke-free workplace ordinance in Hong Kong. Employers with a higher level of knowledge and perceived impact of smoking on companies and from blue-collar companies were more likely to promote SC in workplace. The findings inform future workplace intervention design and policy.

## Abbreviation

aOR: Adjusted odds ratio
CI: Confidence interval
NRT: Nicotine replacement therapy
SC: Smoking cessation
SD: Standard deviation
SHS: Secondhand smoke
